# Deciphering the role of gut microbiota metabolites in bronchiectasis through the perspectives of network pharmacology, Mendelian randomization, and molecular dynamics

**DOI:** 10.1097/MD.0000000000046535

**Published:** 2025-12-19

**Authors:** Qinzhe Zhang, Shang Li, Shaochu Zheng, Jinling Tang, Haizhu Huang, Jiahui Han, Yue Zhou, Yanping Liu, Xiaopu Wu, Jing Luo, Jinliang Kong

**Affiliations:** a Department of Pulmonary and Critical Care Medicine, The First Affiliated Hospital of Guangxi Medical University, Nanning, Guangxi Province, China; b Department of Pulmonary and Critical Care Medicine, The Affiliated Minzu Hospital of Guangxi Medical University, Nanning, Guangxi Province, China.

**Keywords:** bronchiectasis, gut microbiota, Mendelian randomization, metabolites, molecular dynamics, network pharmacology

## Abstract

The role of gut microbiota and their metabolites in respiratory diseases via the “gut-lung axis” has garnered increasing attention, yet their specific mechanisms in bronchiectasis (BIS) remain unclear. This study integrates network pharmacology with bioinformatics approaches, including Mendelian randomization (MR) and molecular dynamics (MD), to systematically investigate the mechanisms of gut microbiota metabolites in BIS pathogenesis and explore potential therapeutic interventions. Intersection targets between gut microbiota metabolites and BIS were screened using network pharmacology. A protein–protein interaction (PPI) network was constructed, and MR combined with MD simulations were employed to validate interactions between core targets and metabolites. A total of 40 metabolite-disease intersection targets were identified, with 17 core genes prioritized. MR analysis revealed a significant protective effect of the peroxisome proliferator-activated receptor gamma (PPARG) gene against BIS (IVW method: β = −0.141, OR = 0.868, *P* = .030). Molecular docking confirmed strong binding affinity of butyrate and 10-keto-12Z-octadecenoic acid to PPARG (affinity: −3.731 and −5.666 kcal/mol, respectively). Drug-likeness and toxicological analyses indicated both compounds possess therapeutic potential, with 10-keto-12Z-octadecenoic acid demonstrating superior properties. MD simulations further validated the stability of metabolite-PPARG complexes. Gut microbiota metabolites mediate protective mechanisms in BIS pathogenesis through PPARG, and 10-keto-12Z-octadecenoic acid emerges as a novel lead compound for treatment. This study provides a theoretical foundation for precision therapy targeting the gut-lung axis.

## 1. Introduction

Bronchiectasis (BIS) is a chronic respiratory disease characterized by abnormal airway structure and recurrent infections. Its pathogenesis is closely associated with chronic inflammation and immune imbalance, where chronic infections, inflammation, mucociliary dysfunction, and airway damage interact to form the core mechanism of disease progression,^[1,2]^ Due to the widespread adoption of imaging technology and increased awareness, the global incidence and prevalence of BIS have risen significantly, imposing a substantial socioeconomic burden.^[3]^ Traditional perspectives attribute its pathogenesis primarily to localized microbial infections and immune abnormalities in the lungs. However, emerging research suggests that gut microbiota and their metabolites play a critical role in the development and progression of BIS through the “gut-lung axis,” a bidirectional communication system.^[4]^ The concept of the gut-lung axis describes a complex interaction network between the gut and lungs mediated by microbes, metabolites, and immune signals.^[5]^ Recent studies have revealed that gut dysbiosis influences pulmonary disease progression via the gut-lung axis, with BIS patients exhibiting significant gut microbiota disturbances that differ markedly from healthy individuals.^[6]^ The gut microbiota participates in the regulation of the gut-lung axis by producing various bioactive metabolites, such as short-chain fatty acids, which may exert protective effects through immune modulation and signaling pathway regulation.^[7]^ Nevertheless, the specific targets and mechanisms of gut microbiota metabolites in BIS remain unclear. Mendelian randomization (MR) is a method to address the study question by inferring causality. This study systematically deciphers the regulatory network of gut microbiota metabolites in BIS by integrating network pharmacology, MR, and molecular dynamics simulations (MDs), offering novel approaches and insights for therapeutic strategies.

## 2. Methods and materials

### 2.1. Identification of metabolites and targets of gut microbiota

The database sources and software used are listed in Table 1. We obtained metabolites of gut microbiota and their human intestinal targets from the gutMGene database. Subsequently, the metabolites were uploaded to the PubChem database to acquire their corresponding SMILES formats. The targets of gut microbiota metabolites were retrieved from the SEA database and STP database, with the species set as “Homo sapiens.” By employing Venn diagrams, the intersecting common targets of gut microbiota metabolites were extracted from the SEA database and STP database.

**Table 1:** Summary of data sources, analytical platforms, and software information.

No	Database, database and analysis platform	Website	Version
1	gutMGene	http://bio-annotation.cn/gutmgene/	–
2	Swiss target prediction	http://www.swisstargetprediction.ch/	–
3	Pubchem	https://pubchem.ncbi.nlm.nih.gov/	–
4	STRING	https://string-db.org	Version:12.0
5	Cytoscape software	https://cytoscape.org/	Cytoscape_v3.8.0
6	OMIM	https://omim.org/	–
7	Genecards	https://www.genecards.org/	–
8	DAVID Bioinformatics	https://david.ncifcrf.gov/tools.jsp	–
9	SwissAMDE	http://www.swissadme.ch/index.php	–
10	ADMETlab 3.0	https://admetlab3.scbdd.com/	–
11	Similarity ensemble approach (SEA)	https://sea.bkslab.org/	–
12	jvenn	https://jvenn.toulouse.inra.fr/app/example.html	–
13	R	https://www.r-project.org/	R.4.4.1
14	GWAS summary data	https://gwas.mrcieu.ac.uk/	–
15	cis-eQTLs	https://www.eqtlgen.org/cis-eqtls.html	–
16	UniProt	https://www.uniprot.org/	–
17	PDB	https://www.rcsb.org/	–
18	Molecular docking	–	Autodock Vina 1.1.2, MGLTools 1.5.7
19	Molecular dynamics simulation	https://www.gromacs.org/	Gromacs2022

GWAS = genome-wide association study.

### 2.2. Identification of disease targets

Using “BIS” as the keyword, disease-related targets of BIS were searched through databases such as Genecard, OMIM, and CTD (URLs of disease databases are listed in Table 1). Venn diagrams were employed to identify the targets of BIS.

### 2.3. PPI network construction and analysis

The intersecting targets between gut microbiota metabolites and BIS targets were uploaded to the STRING database to construct a protein–protein interaction (PPI) network (the URL of STRING can be found in Table 1).

### 2.4. GO and KEGG enrichment analysis

The intersecting genes were subjected to gene ontology (GO) and Kyoto Encyclopedia of genes and genomes (KEGG) enrichment analyses, and the results were visualized. Statistical analysis: GO and KEGG terms meeting the criterion of “*P* < .05” were included for further analysis. Subsequently, “FDR (false discovery rate) < 0.05” was used to identify significant GO and KEGG terms.

### 2.5. Mendelian randomization

Core targets screened through Cytoscape_v3.8.0 were subjected to MR using publicly available GWAS (genome-wide association study) data and eQTL (expression quantitative trait loci) data. Genetic variants (SNPs) strongly associated with the targets were selected, with a threshold of *P*-value < 1 × 10^−5^, followed by linkage disequilibrium (LD) analysis (clumping, *r*² < 0.001, kb = 10,000). Causal effects were evaluated using methods such as inverse variance weighted (IVW), MR-Egger regression, and weighted median. Sensitivity and pleiotropy analyses were conducted using heterogeneity tests (Cochran *Q*), horizontal pleiotropy tests (MR-Egger intercept), and leave-one-out analysis.

### 2.6. Molecular docking and molecular dynamics simulation

The protein structure was retrieved from the PDB database, and after removing redundant structures such as small molecules and water, it was converted into a pdbqt file using MGLTools 1.5.7. The small molecule structure file was downloaded from the PubChem website and processed into a pdbqt file using MGLTools. A docking box was constructed to encompass the entire protein, and molecular docking between the small molecule and the protein was performed using Autodock Vina 1.1.2.

MDs were conducted with the Gromacs2022 program, employing the GAFF force field for small molecules and the AMBER14SB force field with TIP3P water model for proteins. The protein and small molecule ligand files were merged to construct the simulation system for the complex. Simulations were performed under isothermal-isobaric conditions with periodic boundary constraints. During MD simulations, all hydrogen bonds were constrained using the LINCS algorithm with an integration time step of 2 fs. Electrostatic interactions were calculated using the Particle-Mesh Ewald method with a cutoff of 1.2 nm. The non-bonded interaction cutoff was set to 10 Å and updated every 10 steps. The V-rescale temperature coupling method maintained the simulation temperature at 298 K, while the Berendsen method controlled pressure at 1 bar. The system underwent 100 ps of NVT and NPT equilibration at 298 K, followed by 100 ns of production MD simulation for the complex, with conformations saved every 10 ps. Post-simulation analysis was performed using VMD and PyMOL to examine trajectories, while the g_mmpbsa program was employed for MMPBSA binding free energy calculations between the protein and small molecule ligand.

### 2.7. Drug-likeness and toxicity analysis

The gastrointestinal absorption characteristics and drug-likeness of key metabolites identified through the SwissADME platform were analyzed. Metabolites meeting the criteria for gastrointestinal absorption efficiency (GI absorption as high) and drug-likeness (at least two YES parameters in Druglikeness) were selected for further analysis. The toxicity of the identified key metabolites was assessed using the ADMETlab 3.0 platform.

## 3. Results

### 3.1. Identification of gut microbiota metabolite intervention targets for bronchiectasis

**Figure 1.**
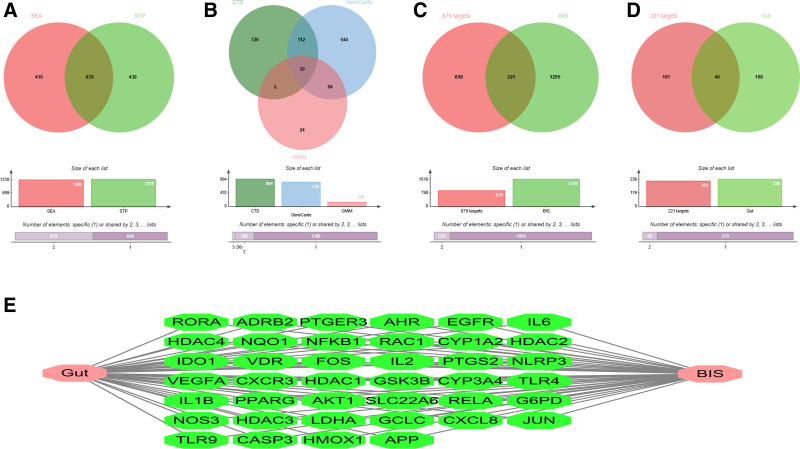
Identification of bronchiectasis-associated genes regulated by gut microbiota metabolites. (A) Overlapping targets of gut microbiota metabolites predicted by both similarity ensemble approach (SEA) and Swiss target prediction (STP). (B) Common targets associated with bronchiectasis (BIS). (C) Intersection between gut microbiota metabolite targets and BIS-associated genes. (D) Shared targets among gut microbiota metabolite-BIS interactions and human intestinal targets. (E) Network visualization of gut microbiota metabolite-target-BIS interactions (Gut-Target-BIS network). BIS = bronchiectasis, SEA = similarity ensemble approach, STP = Swiss target prediction.

First, 251 gut microbiota metabolites and 238 human intestinal targets were obtained from the gutMGene database. Then, we identified 1289 and 1318 targets associated with the 251 gut microbiota metabolites in the SEA and STP databases, respectively. The 879 overlapping targets between SEA and STP were considered the primary targets of the 251 gut microbiota metabolites (Fig. 1A). We retrieved a total of 1516 BIS-related targets from the Genecard, OMIM, and CTD databases (Fig. 1B). After intersecting the 879 common targets of gut microbiota metabolites with these BIS-related targets, we obtained 221 shared targets (Fig. 1C). Finally, a total of 40 targets were identified from the Venn diagram between the 221 shared targets and the 238 intestinal targets (Fig. 1D). Figure 1E illustrates the Gut-Target-BIS network. These results indicate that the 40 targets are responsible for the regulation of BIS by gut microbiota.

### 3.2. PPI network

**Figure 2.**
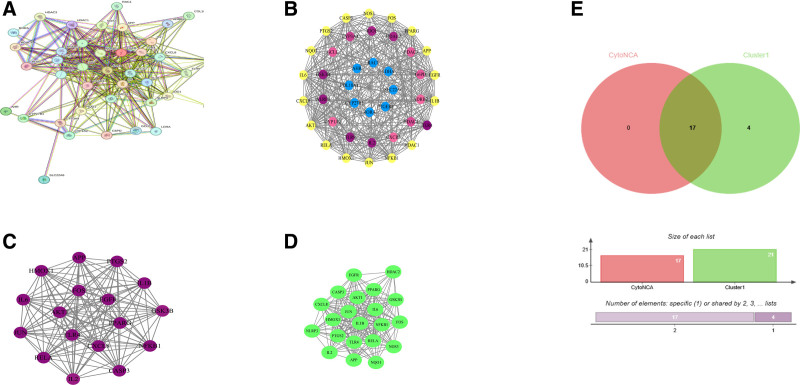
Identification of core genes through PPI network analysis and Cytoscape plugins. (A) PPI network constructed from STRING database. (B) PPI network visualization with hierarchical clustering. (C) Topological analysis using CytoNCA plugin identified 17 core genes. (D) Top-scoring module (Cluster 1) detected by MCODE plugin. (E) Overlap between the 17 core genes (CytoNCA screening) and Cluster 1 genes (MCODE screening). CytoNCA = Cytoscape plugin for network centrality analysis, MCODE = molecular complex detection algorithm, PPI = protein–protein interaction.

Overlapping targets were submitted to the STRING platform for PPI network analysis (Fig. 2A). The network was visualized using Cytoscape 3.8, identifying a total of 40 nodes and 329 edges (Fig. 2B). To further identify the core targets of gut microbiota metabolites in regulating BIS, we conducted an in-depth analysis of target centrality using the Cytoscape plugin for network centrality analysis (CytoNCA). Screening results revealed 17 core genes as the central targets for gut microbiota metabolite regulation in BIS (Table 2, Fig. 2C). To further elucidate the functional significance of these targets, cluster analysis was performed using the MCODE plugin. As shown, cluster 1 (Fig. 2D) comprised 21 nodes and 187 edges, including the 17 core genes identified by CytoNCA (Fig. 2E). These 17 core genes served as the basis for subsequent drug target MR analysis.

**Table 2:** CytoNCA-based identification of 17 core genes regulating gut microbiota metabolites in bronchiectasis.

Name	Betweenness	Closeness	Degree	Eigenvector	LAC	Network
FOS	0.13333333333333333	1	16	0.24410179257392883	14.875	16
PPARG	0.13333333333333333	1	16	0.2441016584634781	14.875	16
APP	0.13333333333333333	1	16	0.24410177767276764	14.875	16
EGFR	0.13333333333333333	1	16	0.24410177767276764	14.875	16
IL1B	0.13333333333333333	1	16	0.24410177767276764	14.875	16
TLR4	0.13333333333333333	1	16	0.24410177767276764	14.875	16
IL2	0	0.9411764705882353	15	0.23045071959495544	14	15
NFKB1	0.13333333333333333	1	16	0.24410177767276764	14.875	16
JUN	0.13333333333333333	1	16	0.24410177767276764	14.875	16
HMOX1	0.13333333333333333	1	16	0.24410177767276764	14.875	16
RELA	0.13333333333333333	1	16	0.24410177767276764	14.875	16
AKT1	0.13333333333333333	1	16	0.24410177767276764	14.875	16
CXCL8	0.13333333333333333	1	16	0.24410177767276764	14.875	16
GSK3B	0	0.9411764705882353	15	0.23045071959495544	14	15
IL6	0.13333333333333333	1	16	0.24410177767276764	14.875	16
PTGS2	0.13333333333333333	1	16	0.24410177767276764	14.875	16
CASP3	0.13333333333333333	1	16	0.24410177767276764	14.875	16

CytoNCA = Cytoscape plugin for network centrality analysis, GWAS = genome-wide association study, LAC = local average connectivity.

### 3.3. GO enrichment analysis and KEGG enrichment analysis

**Figure 3.**
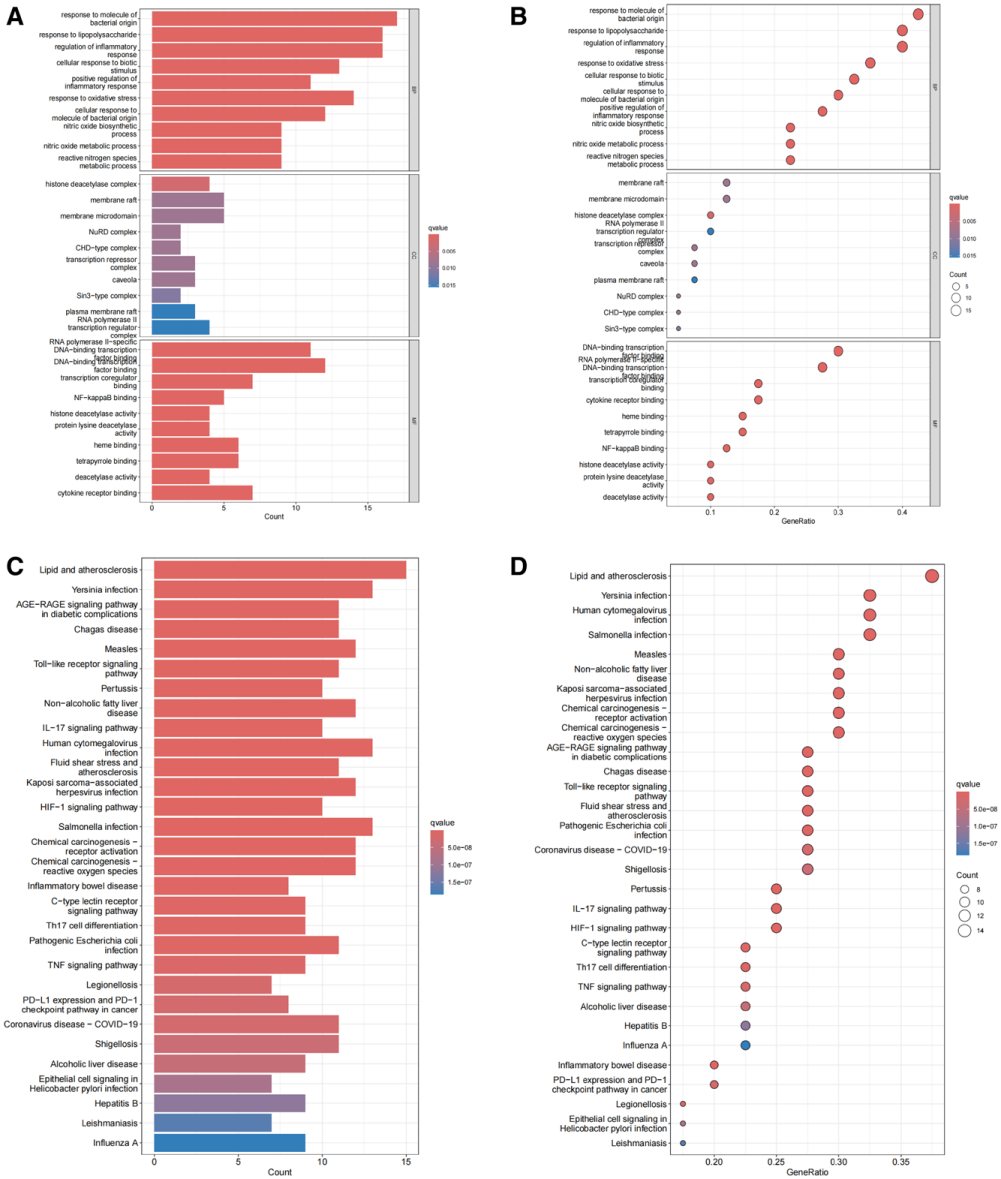
Functional enrichment analysis of core targets linking gut microbiota metabolites to bronchiectasis (BIS). (A) Gene ontology (GO) enrichment bar plot for biological process terms. (B) GO enrichment bubble plot displaying significance (−log10(P)) versus gene ratio. (C) Kyoto Encyclopedia of genes and genomes (KEGG) pathway enrichment bar plot. (D) KEGG enrichment bubble plot highlighting key metabolic and signaling pathways. Note: GO terms are categorized into biological processes (BP), molecular functions (MF), and cellular components (CC). Bubble size represents gene counts; color intensity indicates statistical significance.

GO enrichment analysis revealed that BIS-related genes were significantly enriched in multiple key biological processes and molecular functions (barplot Fig. 3A, bubble Fig. 3B). In the biological process (BP) category, entries related to pathogen response were particularly prominent, including response to molecule of bacterial origin, response to lipopolysaccharide, and regulation of inflammatory response. Additionally, pathways such as response to oxidative stress, nitric oxide biosynthetic process, and reactive nitrogen species metabolic process were significantly enriched, suggesting that oxidative-antioxidant imbalance may play a critical role in disease progression. Molecular function analysis indicated that core targets were primarily involved in transcriptional regulatory mechanisms, such as RNA polymerase II-specific DNA-binding transcription factor binding and NF-kappaB binding, while also being closely associated with functions like histone deacetylase activity and cytokine receptor binding. Cellular component enrichment analysis further demonstrated that target genes were significantly localized to membrane raft, histone deacetylase complex, and RNA polymerase II transcription regulator complex, suggesting that epigenetic regulation and membrane signal transduction may synergistically contribute to pathological mechanisms.

KEGG pathway enrichment analysis (barplot Fig. 3C, bubble Fig. 3D) unveiled a multidimensional signaling network associated with BIS. Infection- and immunity-related pathways dominated, including the Toll-like receptor signaling pathway, IL-17 signaling pathway, and various pathogen infection pathways, indicating that pathogen-host interaction is a critical trigger for the disease. The enrichment of metabolic disease-related pathways, cancer-related pathways (e.g., PD-L1/PD-1 checkpoint pathway), and inflammatory bowel disease pathways suggested that BIS may share molecular-level associations with systemic inflammation and immune dysregulation. The synergistic characteristics of these multiple pathways provide important clues for deciphering the complex molecular mechanisms underlying this disease.

### 3.4. Mendelian randomization

**Figure 4.**
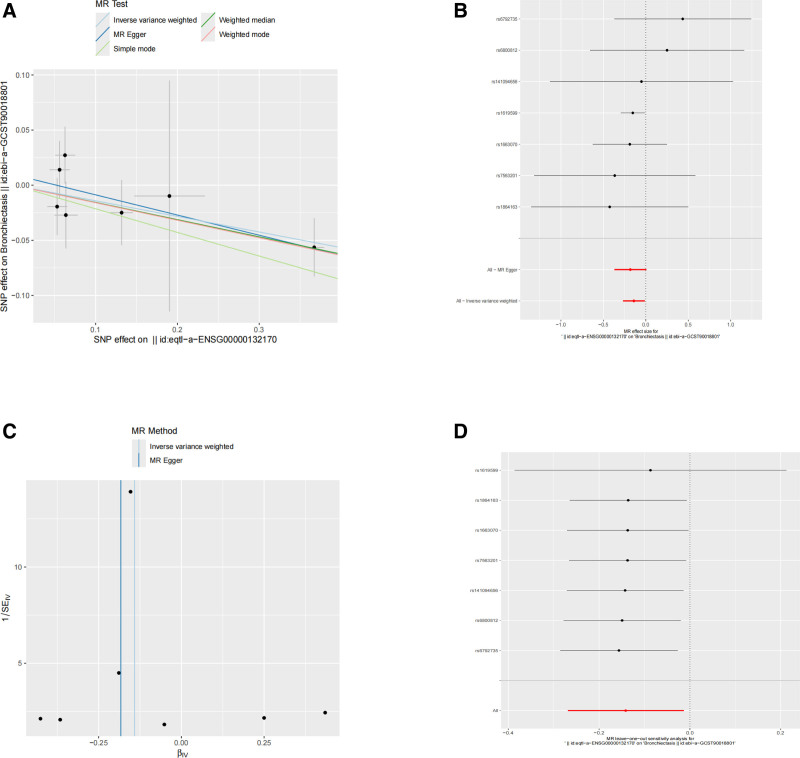
Mendelian randomization (MR) analysis validating causal associations between core genes and bronchiectasis (BIS). (A) Scatter plot of SNP effect sizes on core gene expression (exposure) versus BIS risk (outcome). (B) Forest plot assessing heterogeneity across instrumental variables (IVs). (C) Funnel plot evaluating potential horizontal pleiotropy. (D) Leave-one-out sensitivity analysis testing robustness of causal estimates. IVs = instrumental variables, MR = Mendelian randomization; SNP = single nucleotide polymorphism.

Based on the network centrality analysis of core targets using the CytoNCA plugin in Cytoscape_v3.8.0, this study identified 17 core gene targets. Further two-sample MR analysis was conducted using eQTL data (Table 3) from the GWAS database and genetic association data of BIS. The results revealed a significant negative causal relationship between the metabolite target gene peroxisome proliferator-activated receptor gamma (PPARG) (eqtl-a-ENSG00000132170) and BIS risk (ebi-a-GCST90018801), suggesting that activation of the PPARG gene may reduce disease risk through metabolic regulatory pathways. The inverse-variance weighted (IVW) method showed that each unit increase in PPARG gene expression was significantly associated with a 14.1% reduction in BIS risk (β = −0.141, OR = 0.868, 95% CI: 0.764–0.986, *P* = .030). The weighted median method demonstrated consistent effect direction with enhanced significance (β = −0.156, *P* = .023). MR-Egger regression, though not statistically significant, exhibited consistent effect direction (β = −0.183, *P* = .113), indicating a low risk of potential weak instrument variable bias. Heterogeneity *Q* tests detected no significant heterogeneity in either the IVW model (*Q* = 3.366, df = 6, *P* = .762) or the MR-Egger model (*Q* = 3.014, df = 5, *P* = .698). The MR-Egger intercept test showed no significant deviation (intercept = 0.0095, *P* = .579). The MR-PRESSO global test found no outlier instrument variables (global test *P* = .836), and the original IVW estimate remained robust (β = −0.14, *P* = .027). Leave-one-out analysis confirmed that the causal effect estimates remained stable after excluding any single instrument variable, indicating that the results were not driven by a single SNP. In summary, the MR analysis supports the protective role of metabolites against BIS through the PPARG gene regulatory pathway (detailed results shown in MR visualization results Fig. 4A–D).

**Table 3:** Mendelian randomization dataset characteristics and instrumental variable details.

GWAS summary data	Name	ID	Trait	Population	nsnp	Sample_size
Exposure data	FOS	eqtl-a-ENSG00000170345	ENSG00000170345	European	17,275	31,684
	PPARG	eqtl-a-ENSG00000132170	ENSG00000132170	European	19,079	31,684
	APP	eqtl-a-ENSG00000142192	ENSG00000142192	European	18,927	31,470
	EGFR	NA	NA	NA	NA	NA
	IL1B	eqtl-a-ENSG00000125538	ENSG00000125538	European	17,485	31,684
	TLR4	eqtl-a-ENSG00000136869	ENSG00000136869	European	18,916	31,684
	IL2	NA	NA	NA	NA	NA
	NFKB1	eqtl-a-ENSG00000109320	ENSG00000109320	European	18,293	30,935
	JUN	eqtl-a-ENSG00000177606	ENSG00000177606	European	18,452	31,470
	HMOX1	eqtl-a-ENSG00000100292	ENSG00000100292	European	20,094	31,684
	RELA	eqtl-a-ENSG00000173039	ENSG00000173039	European	16,253	31,684
	AKT1	eqtl-a-ENSG00000142208	ENSG00000142208	European	20,441	30,721
	CXCL8	eqtl-a-ENSG00000169429	ENSG00000169429	European	17,359	25,646
	GSK3B	eqtl-a-ENSG00000082701	ENSG00000082701	European	18,497	31,684
	IL6	eqtl-a-ENSG00000136244	ENSG00000136244	European	19,276	30,765
	PTGS2	eqtl-a-ENSG00000073756	ENSG00000073756	European	18,166	31,470
	CASP3	eqtl-a-ENSG00000164305	ENSG00000164305	European	20,415	31,684
Outcome data	Bronchiectasis	ebi-a-GCST90018801	Bronchiectasis	European	24,189,609	443,151

nsnp = number of SNPs.

### 3.5. Molecular docking and molecular dynamics simulation

**Figure 5.**
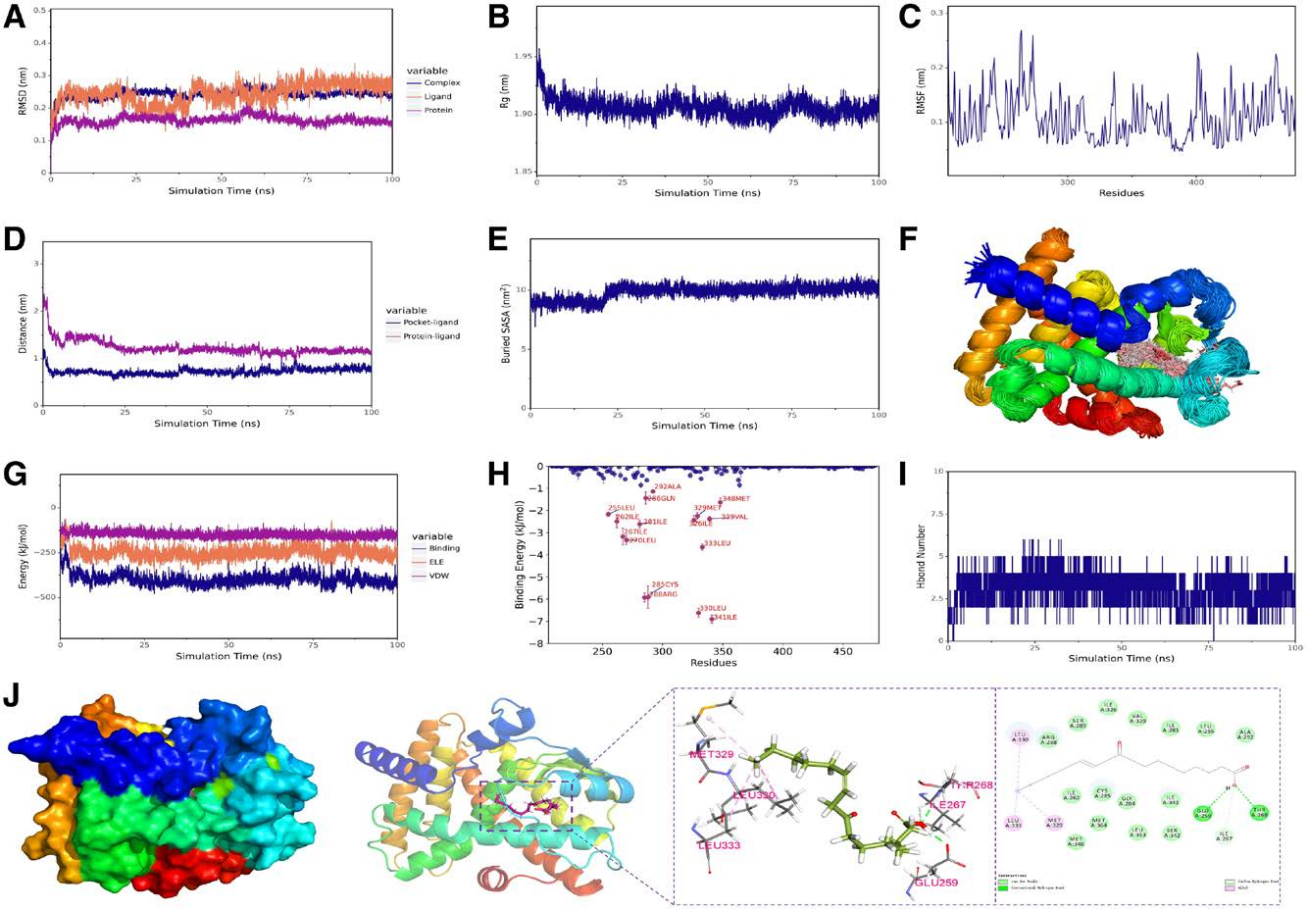
The molecular dynamics simulation results of metabolite 10-keto-12Z-octadecenoic acid with PPARγ. (A) RMSD of the complex, protein, and small molecule ligand Ligand. (B) Rg of the complex; (C) RMSF of the protein in the complex. (D) Distance between the protein and small molecule binding site (Dock site-ligand). (E) Buried surface area (Buried SASA) between the small molecule and protein. (F) Superimposition of simulated conformations. (G) Binding energy between the small molecule and protein (VDW and ELE ). (H) Amino acid binding energy contribution. (I) Number of hydrogen bonds (Hbond number). (J) Interactions between the protein and small molecule. VDW = Van der Waals, ELE = electrostatic interactions.

We performed molecular docking of PPARG gene-targeted metabolites using AutoDock Vina v1.2.3, with the results showing that the optimal binding energies for Butyrate and 10-keto-12Z-octadecenoic acid were −3.731 and −5.666 kcal/mol, respectively. We conducted MDs of 10-keto-12Z-octadecenoic acid and PPARγ using the Gromacs2022 program. As shown in Figure 5A, the RMSD of the complex structure gradually stabilized during the simulation, indicating the progressive stabilization of the complex structure. Figure 5B reveals that the Rg of the complex gradually stabilized over the simulation, further confirming the structural stability. Figure 5C presents RMSF analysis, which helps identify flexible and rigid regions within the molecule. Figure 5D demonstrates that the distances between the small molecule and the protein center, as well as the binding site, gradually stabilized, indicating stable binding of the small molecule to the protein binding site. Figure 5E shows that the Buried SASA gradually stabilized, reflecting the stable contact area between the small molecule and the protein. The simulated trajectories were processed, and the superimposed conformations are displayed in Figure 5F. Figure 5G indicates that the Van der Waals and electrostatic interactions within the complex gradually stabilized during the simulation, signifying the progressive stabilization of the binding between the small molecule and the protein. The high degree of small molecule superposition suggests consistent binding to the protein. Here, VDW represents van der Waals and hydrophobic interactions, while ELE denotes electrostatic interactions.

Considering solvation energy and integrating RMSD, Rg, Distance, Buried SASA, and interaction energy, we selected the stable complex trajectory and calculated the binding energy-related terms using the MM-PBSA (Molecular Mechanics-Poisson Boltzmann Surface Area) method. The resulting ΔEMMPBSA between the small molecule and the protein was −99.822 ± 3.289 kJ/mol, indicating high binding energy and affinity. Decomposition of ΔEMMPBSA identified key amino acids contributing to the overall binding energy, with significant residues such as ILE-341 and LEU-330 highlighted in Figure 5H. Hydrogen bonds, which correlate with electrostatic interactions, reflect their strength, Figure 5I shows that the number of hydrogen bonds between the small molecule and the protein fluctuated between 2 and 4.

A stable simulation conformation was selected for structural and interaction analysis, as illustrated in Figure 5J. The amino acids THR-268 and GLU-259 formed hydrogen bonds with the small molecule, while LEU-330, LEU-333, and MET-329 engaged in hydrophobic (Alkyl) interactions. Additionally, LEU-353, ARG-288, and ALA-292 participated in van der Waals interactions with the small molecule.

In conclusion, the simulation results indicate that 10-keto-12Z-octadecenoic acid exhibits excellent conformational adaptability and binding persistence, validating its robust binding stability and kinetic adaptability. These findings provide theoretical support at the structural and energetic levels for its potential as a PPARγ modulator and lay a solid molecular foundation for subsequent functional studies and pharmacophore optimization.

### 3.6. Drug-likeness and toxicity evaluation

A systematic assessment of two PPARG-targeted metabolites based on the Drug-likeness and multi-dimensional toxicity evaluation system revealed the following (Table 4).

**Table 4:** Drug-likeness and multidimensional toxicity evaluation of 2 PPARG-targeted metabolites.

Compound	GI absorption	Druglikeness						Toxicity			
		Lipinski	Ghose	Veber	Egan	Muegge	Bioavailability score	hERG Blockers	H-HT	DILI	Carcinogencity
Butyrate	High	Yes	No	Yes	Yes	No	0.55	Excellent	Medium	Excellent	Medium
10-Keto-12Z-octadec-enoic acid	High	Yes	Yes	No	Yes	No	0.85	Excellent	Excellent	Excellent	Excellent

DILI = drug induced liver injury, GI = gastrointestinal, H-HT = human hepatotoxicity, PPARG = peroxisome proliferator-activated receptor gamma.

Butyrate complies with Lipinski’s Rule of Five as well as the Veber and Egan rules, with a bioavailability score of 0.55, indicating moderate oral absorption potential. It exhibits minimal cardiac toxicity (hERG Blockers) and drug-induced liver injury risk (both rated “excellent”), but shows moderate human hepatotoxicity (H-HT: medium) and potential carcinogenicity (Carcinogenicity: medium).

10-Keto-12Z-octadecenoic acid satisfies the Lipinski, Ghose, and Egan rules, achieving a bioavailability score of 0.85, significantly outperforming butyrate, which suggests superior intestinal absorption and systemic distribution properties. All toxicity indicators (hERG Blockers, H-HT, drug-induced liver injury, carcinogenicity) are rated “excellent,” indicating extremely low risks of cardiotoxicity, hepatotoxicity, and carcinogenic potential.

## 4. Discussion

Stratification and personalized management of BIS patients are critical for improving clinical outcomes, necessitating a paradigm shift from uniform treatment to precision frameworks based on comprehensive assessment of disease severity (e.g., BSI/FACED scores), exacerbation/mortality risk, and pathogen profiles – particularly drug-resistant Pseudomonas aeruginosa. This stratification strategy enables effective identification of high-risk patients (requiring intensive intervention) versus low-risk patients (avoiding overtreatment), guiding therapeutic goal-setting, surveillance intensity, and targeted treatment decisions – including precision selection of long-term macrolides, inhaled antibiotics, or acute-phase antimicrobial regimens, alongside optimization of non-antibiotic therapies such as airway clearance and anti-inflammatory agents. Continuous monitoring of symptoms, pathogens, and pulmonary function facilitates dynamic treatment adjustments through closed-loop management, ultimately aiming to significantly reduce exacerbations, slow lung function decline, lower mortality risk, and enhance quality of life. This study systematically elucidates, for the first time, the molecular mechanisms by which gut microbiota metabolites regulate BIS through the “gut-lung axis” by integrating network pharmacology, MR, and MD techniques. The findings not only validate the multi-target regulatory properties of gut microbiota metabolites but also identify key effector targets through causal inference methods, providing a crucial theoretical foundation for subsequent translational research.

First, network pharmacological analysis revealed that the regulatory network composed of 40 intersection targets between gut microbiota metabolites and BIS exhibits significant functional heterogeneity. The 17 core targets (such as PPARG, AKT1, IL6, FOS, NFKB1, HMOX1, GSK3B, etc) identified from the PPI network are predominantly enriched in biological processes including response to molecule of bacterial origin, response to lipopolysaccharide, regulation of inflammatory response, and response to oxidative stress. This finding aligns closely with the pathological features of BIS – studies have confirmed that infections further activate neutrophils, prompting them to release neutrophil serine proteases (such as elastase NE and cathepsin G), which directly degrade elastin and extracellular matrix (ECM) in lung tissue, damaging bronchial wall structure.^[8]^ Oxidative stress activates NADPH oxidase and PAD4 (peptidylarginine deiminase 4), promoting chromatin decondensation and NETs release,^[9]^ The DNA scaffold and attached proteins (e.g., elastase, myeloperoxidase) of NETs can directly damage the airway epithelial barrier, increasing permeability and leading to mucus accumulation and bacterial colonization,^[10]^ Proteases such as NE released by NETs exceed the inhibitory capacity of local antiproteases (e.g., α1-antitrypsin), accelerating ECM degradation and bronchial wall destruction.^[11]^ Infection, inflammation, and oxidative stress collectively contribute to bronchial wall structural damage, forming a vicious cycle. Notably, the significant enrichment of IL-17, Toll-like receptor, and TNF signaling pathways in KEGG enrichment analysis suggests that gut microbiota metabolites may influence the pulmonary immune microenvironment by regulating Th17/Treg immune imbalance and pathogen pattern recognition receptor activation.

Through MR analysis, we have for the first time verified the protective effect of the PPARG gene on BIS at the causal association level (IVW method β = −0.141, OR = 0.868, 95% CI: 0.764–0.986, *P* = .030). PPARG (peroxisome proliferator activated receptor gamma) encodes the peroxisome proliferator-activated receptor γ protein (PPARγ), which is a member of the nuclear receptor superfamily and functions as a ligand-dependent transcription factor, playing a central role in various physiological and pathological processes. PPARγ exhibits multi-target regulatory potential in respiratory diseases, particularly demonstrating significant therapeutic value in immunometabolic regulation (e.g., macrophage polarization, mitochondrial function) and airway remodeling inhibition.^[12–14]^ Moreover, studies have clearly confirmed that PPARγ expression is lower in the airways of non-CF BIS subjects, and this low-level PPARγ expression is intrinsically linked to the presence of high levels of Pseudomonas aeruginosa.^[15]^

Based on the molecular docking and dynamics simulation results, the best affinity of butyrate and 10-keto-12Z-octadecenoic acid to PPARG reached −3.731 kcal/mol and −5.666 kcal/mol, respectively. During the 100 ns simulation, its excellent binding stability and dynamic adaptability were verified, indicating that 10-keto-12Z-octadecenoic acid may regulate PPARG’s transcriptional activity by directly binding to its ligand-binding domain.

As a short-chain fatty acid produced by gut microbiota metabolism, butyrate plays a pivotal role in the gut-lung axis. Studies have demonstrated that butyrate can alleviate MRSA-induced pulmonary inflammation by improving gut microbiota and promoting M2 polarization of alveolar macrophages.^[16]^ During influenza virus infection, decreased butyrate levels due to gut dysbiosis are associated with disease severity.^[17]^ In a golden hamster model of SARS-CoV-2 infection, butyrate supplementation exhibited tissue damage-mitigating effects, providing direct evidence for its protective role in viral infections and suggesting its potential as an adjunctive therapeutic strategy for COVID-19.^[18]^ Butyrate significantly reduces MUC5AC gene expression in the human airway epithelial cell line A549, indicating its regulatory effect on mucus secretion.^[19]^ This discovery offers new insights for treating mucus hypersecretion in chronic airway diseases. As a multifunctional bioactive molecule, butyrate exerts potential therapeutic effects in the pathological process of BIS through anti-inflammatory, immunomodulatory, and gut-lung axis mechanisms.^[20]^ Future clinical studies are needed to further validate its efficacy, particularly for patients with different etiologies (e.g., post-infectious, idiopathic) and varying disease severities.

It is noteworthy that drug-likeness analysis revealed 10-keto-12Z-octadecenoic acid exhibits high oral bioavailability (Bioavailability score = 0.85) and safety profile (no hepatotoxicity or carcinogenicity). Its molecular weight (296.44 g/mol) and polar surface area (54.37 Å^2^) both comply with Lipinski rule, indicating promising druggability potential. In contrast, butyrate (oral bioavailability score = 0.55), despite demonstrating definitive anti-inflammatory activity with its molecular weight (110.09 g/mol) and polar surface area (40.13 Å^2^), may face clinical application limitations due to its short half-life and low blood-brain barrier penetration. The long-chain structure of 10-keto-12Z-octadecenoic acid could potentially provide more sustained pharmacokinetic properties. The discovery of this novel metabolite, 10-keto-12Z-octadecenoic acid, offers a new direction for developing gut microbiota-based therapeutics for BIS.

## 5. Conclusions

This study systematically elucidated the multi-target network through which gut microbiota metabolites regulate BIS via multi-omics integrative analysis. PPARG emerged as the core target for gut microbiota metabolite intervention in BIS, with MR providing the first evidence of a significant negative causal association between PPARG genetic variants and BIS risk. Although butyrate exhibits anti-inflammatory activity, its moderate bioavailability (0.55) and potential carcinogenic risk may limit its direct clinical application, necessitating formulation improvements (e.g., nano-encapsulation or prodrug design) to enhance stability and targeting.^[21]^ In contrast, 10-keto-12Z-octadecenoic acid demonstrates superior druggability potential, characterized by high bioavailability (0.85), comprehensively low toxicity (all “excellent”), and compliance with key drug-likeness rules (Lipinski, Ghose), positioning it as a promising lead compound for developing novel BIS therapeutics.

This study has certain limitations: First, the ethnic bias in GWAS data (primarily derived from European populations) may affect the generalizability of the MR results. This limitation arises from ancestry-dependent variations in genetic architecture, including divergent linkage disequilibrium (LD) patterns, allele frequencies, and gene-environment interactions. To address this challenge, we propose implementing a multidimensional integrative strategy encompassing: enhanced genotype imputation techniques (e.g., cross-population genotype imputation and development of ancestry-specific reference panels); innovative statistical modeling approaches (including trans-ancestry meta-analysis, Bayesian multi-ancestry frameworks, and LD-adjusted association optimization); computational efficiency advancements (such as distributed GWAS computing architectures and GPU-accelerated association algorithms); and consortium-level research design protocols (featuring global collaborative networks and standardized ethical frameworks for equitable data sharing), among other critical initiatives; second, the molecular dynamics findings require validation of target binding specificity through in vitro experiments; finally, the concentration gradient effects of gut microbiota metabolites and their spatiotemporal dynamic transmission mechanisms in the gut-lung axis still need further exploration.

Future research should validate the in vivo pharmacological effects of these metabolites using animal models and explore the activation effects of gut microbiota-targeted regulation strategies on the PPARG pathway in BIS patients. This study provides a novel perspective for precision therapy based on the gut-lung axis and also lays a methodological foundation for microbiome intervention research in other chronic respiratory diseases.

## Author contributions

**Conceptualization:** Jinling Tang, Jinliang Kong.

**Data curation:** Qinzhe Zhang, Shang Li, Shaochu Zheng, Haizhu Huang.

**Formal analysis:** Qinzhe Zhang.

**Methodology:** Qinzhe Zhang, Jiahui Han.

**Resources:** Jinliang Kong.

**Software:** Shang Li, Jiahui Han, Yue Zhou.

**Supervision:** Yue Zhou, Yanping Liu.

**Validation:** Xiaopu Wu.

**Writing – original draft:** Qinzhe Zhang.

**Writing – review & editing:** Jing Luo, Jinliang Kong.

## References

[1] Perea L Faner R Chalmers JD Sibila O. Pathophysiology and genomics of bronchiectasis. Eur Respir Rev. 2024;33:240055. 38960613 10.1183/16000617.0055-2024 PMC11220622

[2] Azoicai A Lupu A Alexoae MM. Lung microbiome: new insights into bronchiectasis’ outcome. Front Cell Infect Microbiol. 2024;14:1405399. 38895737 10.3389/fcimb.2024.1405399 PMC11183332

[3] Choi H McShane PJ Aliberti S Chalmers JD. Bronchiectasis management in adults: state of the art and future directions. Eur Respir J. 2024;63:2400518. 38782469 10.1183/13993003.00518-2024 PMC11211698

[4] Wang WW Mao B Liu Y. Altered fecal microbiome and metabolome in adult patients with non-cystic fibrosis bronchiectasis. Respir Res. 2022;23:317. 36403022 10.1186/s12931-022-02229-w PMC9675243

[5] Cheng Y Hu G Deng L Zan Y Chen X. Therapeutic role of gut microbiota in lung injury-related cognitive impairment. Front Nutr. 2024;11:1521214. 40017811 10.3389/fnut.2024.1521214 PMC11867030

[6] Wang LL Shen X Xie Y. A gut Eggerthella lenta-derived metabolite impairs neutrophil function to aggravate bacterial lung infection. Sci Transl Med. 2025;17:eadq4409. 40009694 10.1126/scitranslmed.adq4409

[7] Rastogi S Mohanty S Sharma S Tripathi P. Possible role of gut microbes and host’s immune response in gut-lung homeostasis. Front Immunol. 2022;13:954339. 36275735 10.3389/fimmu.2022.954339 PMC9581402

[8] Chalmers JD Metersky M Aliberti S. Neutrophilic inflammation in bronchiectasis. Eur Respir Rev. 2025;34:240179. 40174958 10.1183/16000617.0179-2024 PMC11962982

[9] Lee HT Lin CS Liu CY Chen P Tsai CY Wei YH. Mitochondrial plasticity and glucose metabolic alterations in human cancer under oxidative stress-from viewpoints of chronic inflammation and neutrophil extracellular traps (NETs). Int J Mol Sci. 2024;25:9458. 39273403 10.3390/ijms25179458 PMC11395599

[10] Jo A Kim DW. Neutrophil extracellular traps in airway diseases: pathological roles and therapeutic implications. Int J Mol Sci. 2023;24:5034. 36902466 10.3390/ijms24055034 PMC10003347

[11] Chalmers JD Mall MA Chotirmall SH. Targeting neutrophil serine proteases in bronchiectasis. Eur Respir J. 2025;65:2401050. 39467608 10.1183/13993003.01050-2024 PMC11694565

[12] He S Tian R Zhang X. PPARgamma inhibits small airway remodeling through mediating the polarization homeostasis of alveolar macrophages in COPD. Clin Immunol. 2023;250:109293. 36934848 10.1016/j.clim.2023.109293

[13] Ferreira BL Ramirez-Moral I Otto NA Salomao R de Vos AF van der Poll T. The PPAR-gamma agonist pioglitazone exerts proinflammatory effects in bronchial epithelial cells during acute Pseudomonas aeruginosa pneumonia. Clin Exp Immunol. 2022;207:370–7. 35553637 10.1093/cei/uxab036 PMC9113127

[14] Li G Zhang Y Jiang H. PPARG/SPP1/CD44 signaling pathway in alveolar macrophages: mechanisms of lipid dysregulation and therapeutic targets in idiopathic pulmonary fibrosis. Heliyon. 2025;11:e41628. 39866448 10.1016/j.heliyon.2025.e41628 PMC11761845

[15] Burr LD Rogers GB Chen AC. PPARγ is reduced in the airways of non-CF bronchiectasis subjects and is inversely correlated with the presence of Pseudomonas aeruginosa. PLoS One. 2018;13:e0202296. 30114278 10.1371/journal.pone.0202296 PMC6095532

[16] Zhao Y Sun H Chen Y. Butyrate protects against MRSA pneumonia via regulating gut-lung microbiota and alveolar macrophage M2 polarization. mBio. 2023;14:e0198723. 37754570 10.1128/mbio.01987-23 PMC10653920

[17] Chollet L Heumel S Deruyter L. Faecalibacterium duncaniae as a novel next generation probiotic against influenza. Front Immunol. 2024;15:1347676. 38590519 10.3389/fimmu.2024.1347676 PMC11000806

[18] Yu H Yuan L Yan Z. Butyrate protects against SARS-CoV-2-induced tissue damage in golden hamsters. Int J Mol Sci. 2023;24:14191. 37762492 10.3390/ijms241814191 PMC10532055

[19] Kim HS Kim B Holzapfel WH Kang H. Lactiplantibacillusplantarum APsulloc331261 (GTB1()) promotes butyrate production to suppress mucin hypersecretion in a murine allergic airway inflammation model. Front Microbiol. 2023;14:1292266. 38449878 10.3389/fmicb.2023.1292266 PMC10915089

[20] Correa RO Castro PR Moser R. Butyrate: connecting the gut-lung axis to the management of pulmonary disorders. Front Nutr. 2022;9:1011732. 36337621 10.3389/fnut.2022.1011732 PMC9631819

[21] Lauterbach AL Slezak AJ Wang R. Mannose-decorated co-polymer facilitates controlled release of butyrate to accelerate chronic wound healing. Adv Healthc Mater. 2023;12:e2300515. 37503634 10.1002/adhm.202300515 PMC11468131

